# *In Situ* Reduction and Fixation of the Anterior Medial Fenestration Approach for Femoral Head Fracture

**DOI:** 10.1111/os.12578

**Published:** 2019-11-25

**Authors:** Zheng‐hao Wang, Kai‐nan Li, Ping Zhao, Er‐dong Chen, Jiang Zheng

**Affiliations:** ^1^ Affiliated Hospital of Chengdu University, Department of Orthopaedic Surgery Chengdu China

**Keywords:** Anterior medial, Approach, Femoral head, Fenestration, Fracture

## Abstract

**Objective:**

To investigate the feasibility and clinical application of the anterior medial fenestration approach in the treatment of Pipkin type I and II femoral head fractures.

**Methods:**

The hips of two anti‐corrosion adult specimens treated with formalin were dissected and the anatomical structures and directional characteristics of the anterior medial main muscles, ligaments, blood vessels, and nerves were observed. The anterior medial fenestration approach was performed on bilateral hips of four fresh frozen specimens to determine the required pulling direction of the stripped muscles and ligaments during surgery. In addition, the vascular and nerve traction protection directions exposed in the approach were observed and analyzed. The feasibility of this approach was assessed, and the operative approach and critical anatomical depth were measured. We retrospectively analyzed 12 patients with Pipkin type I and II femoral head fractures who underwent *in situ* reduction and fixation by anterior medial fenestration in our hospital from February 2016 to April 2018. The study group included 3 men and 9 women aged 37–59 years (mean, 48.50 years). There were 8 cases of Pipkin type I and 4 cases of Pipkin type II. The operation time, blood loss, fracture healing time, last Thompson–Epstein evaluation, and Harris score were recorded.

**Results:**

A total of 8 fresh frozen specimens from 4 bilateral hips were exposed by anterior medial fenestration. The upper boundary of observation fenestration was the pubic body (anterior acetabulum), and the outer upper boundary was the iliacus and the psoas muscle. The lateral boundary was the rectus femoris and the femoral vessels, while the lower boundary was the transverse branch of the medial femoral circumflex artery and vein. The medial boundary was the pubis muscle, the short adductor muscle, and the long adductor muscle. The pubofemoral and iliofemoral ligaments were observed during fenestration. By cutting open the joint capsule and moving the hip joint, the four quadrants of the femoral head can be exposed. Twelve patients with femoral head fractures who were treated with anterior medial fenestration underwent *in situ* reduction and fixation. The operation time was 96.25–118.75 min (median, 100 min), and the blood loss was 115.00 ± 22.76 mL. The follow‐up time was 18.58 ± 4.48 months, and the fracture healing time was 144.17 ± 14.53 days. The last Thompson–Epstein evaluation was excellent in 6 cases, good in 4 cases, and fair in 2 cases; the excellent and good rate was 83.33%. Finally, the last Harris score was 85.08 ± 5.73 points.

**Conclusions:**

The upper and lower boundaries, inner and outer boundaries, and rear anatomical structure of the anterior medial fenestration approach were defined. The movable hip joint can expose the four quadrants in front of the femoral head in this fenestration. Anterior medial fenestration *in situ* reduction and fixation surgery is feasible and safe for the treatment of Pipkin type I and II femoral head fractures.

## Introduction

Femoral head fractures are usually associated with traumatic hip dislocation and are reported to account for approximately 5%–15% of all hip dislocations^1^. Indeed, Kelly and Lipscomb reported a frequency of 2 cases of femoral head fractures per million people per year^2^. Although high‐speed motor vehicles and industrial advances have led to an increase in the incidence of femoral head fractures, it is still a rare injury^3^. The most common cause of femoral head fractures is high‐energy traffic accidents^4^; the incidence reported by Pipkin^5^ was approximately 92% (23/25), while Kelly and Yarbrough reported an incidence of 92.6% (25/27)^6^.

There are many surgical approaches to femoral head fractures; however, the choice of surgical approach is still controversial. Commonly used approaches include the posterolateral Kocher–Langenbeck approach, the anterolateral Smith–Petersen approach, the anterior Hueter approach, the lateral Watson–Jones approach, the greater trochanter osteotomy approach, and the Ganz approach. The advantage of the anterior approach is that the femoral head is well exposed, and the rate of femoral head necrosis is low. Stannard *et al*. analyzed 26 cases of femoral head necrosis and found that the posterior approach had a higher probability of femoral head necrosis than the anterior approach^7^. However, the disadvantage of the anterior approach is that the probability of heterotopic ossification increases^8^. The advantage of the posterior approach is that a posterior wall injury of the acetabulum is better exposed, and a sciatic nerve injury can be detected; however, the disadvantage is that it is difficult to reset and fix the bone fragments on the anterior side of the femoral head, and the risk of femoral head necrosis increases. Swiontkowski *et al*. recommended anterior surgery, because the blood loss and operative time in the anterior side were reduced relative to the use of the posterior approach. Furthermore, the femoral head was more widely exposed, and there was no difference in terms of the excellent rate of hip function^9^. Different approaches were selected for different fracture types, so as to achieve full exposure and reduction, reduce the impact on the blood supply to the femoral head, and reduce the occurrence of ectopic ossification, osteoarthritis, avascular necrosis of the femoral head, and other related complications^10^. For Pipkin type I and II femoral head fractures with anterior or anteromedial fragments, the S‐P anterolateral approach, the Hueter anterior approach (a vertical incision by the S‐P method), or the Watson–Jones lateral approach are preferred^11^. When the femoral head fracture is accompanied by posterior dislocation of the joint, the posterior joint capsule is severely damaged, and the anterior joint capsule is further damaged when the anterior approach is selected. At the same time, the S‐P and Watson–Jones approaches are closer to lateral, and the exposure of the fracture block of the anterior medial femoral head is poor. The S‐P approach has a large incision exposure range and cutting of the rectus femoris muscle is necessary. In addition, the lateral circumflex femoral artery often needs to be destroyed, and the rate of heterotopic ossification is higher than that of the posterior approach. The K‐L approach is a posterior approach, and the risk of femoral head necrosis is higher than that of the anterior approach. Great trochanteric osteotomy through use of the Ganz approach carries a risk of secondary fracture nonunion of the great trochanter and heterotopic ossification.

In addition, the entire joint capsule needs to be opened during surgery, which may lead to injury of the medial femoral circumflex femoral artery and the round ligament. The above approaches are characterized by extensive exposure and trauma, and require complete incision of the articular capsule and the round ligament. It is necessary to dissociate avulsion fracture pieces completely and then fix them when cutting and reducing the fracture pieces of the head; this method is not conducive to fracture healing. This paper attempts to: (i) introduce a new anteromedial fenestration approach, which can alter the position of the affected limb during surgery, expose the femoral head, and achieve *in‐situ* reduction and fixation of the fracture; (ii) through a study of anatomy, explore the feasibility of this approach; and (iii) based on this theory, explore the clinical curative effect on Pipkin type I and II femoral head fracture cases.

## General Data and Methods

### *Anatomic Study of the Anteromedial Approach*

Two formalin‐treated adult bilateral hip specimens were dissected, including two on the right and two on the left. The specimens were from 1 man, aged 75 years, and 1 woman, aged 68 years. We observed the anteromedial anatomy of the hip and determined the appropriate incision range. The main vascular and nerve branches and their exposure, and the start and end points of muscles and ligaments were observed to clarify the surgical approach.

We dissected the bilateral hips of four fresh frozen cadavers, four on the right and four on the left. Three cadavers were female, and one was male; the age range was 67–85 years (mean, 76.25 years). None of the cadavers underwent hip surgery; the cadavers were dissected in the supine position, and the anteromedial fenestration approach was used to simulate surgery, to determine the traction direction of the dissected muscles needed during the operation. We observed and analyzed the protection direction of the vascular and nerve traction revealed in this approach and evaluated the feasibility of this approach. According to Malizos *et al*., the femoral head was divided into eight quadrants^12^ (front inner lower, front inner upper, front outer lower, front outer upper, lower inner lower, lower inner upper, lower outer upper, and lower outer lower) according to the sagittal plane, coronal plane, and cross‐section.

### *Measurement of Surgical Approach Depth*

The straight‐line distance from the lower edge of the femoral head to the skin of the anteromedial approach was denoted as S_1_.

### *Important Anatomical Depth*

(i) S_2_ represents the straight‐line distance from the medial circumflex femoral artery to the incision skin; (ii) The vertical distance between the plane of the Lesser trochanter and the plane of the femoral artery is denoted by S_3_.

### *Plane Depth of Fenestration*

The linear distance from the lesser trochanter to the incision skin is denoted by S_4_.

### *Retrospective Analysis of Clinical Data from the Anteromedial Approach*

#### *Inclusion and Exclusion Criteria and General Data*

A retrospective analysis of femoral head fracture cases treated by the anteromedial approach in our hospital from February 2016 to April 2018 was performed. The inclusion criteria were as follows: (i) patients with Pipkin type I or II femoral head fractures; (ii) anteromedial fenestration approach for the treatment of femoral head fracture; (iii) patients were followed up for more than 1 year; and (iv) imaging data and case data are complete. The exclusion criteria were as follows: (i) history of hip surgery; (ii) patients with Pipkin type III or IV femoral head fractures; (iii) patients with femoral head fractures that were selected for conservative treatment; and (iv) lost to follow up and incomplete cases.

A total of 12 cases met the inclusion criteria, of which 3were male and 9 were female; the age range was 37–59 years (mean, 48.50 years). There were 8 Pipkin type I cases and 4 Pipkin type II cases. With regards to the injury mechanism, 10 cases were a result of traffic injury and 2 cases were the result of high fall injuries.

#### *Anteromedial Fenestration Approach*

**Figure 1:**
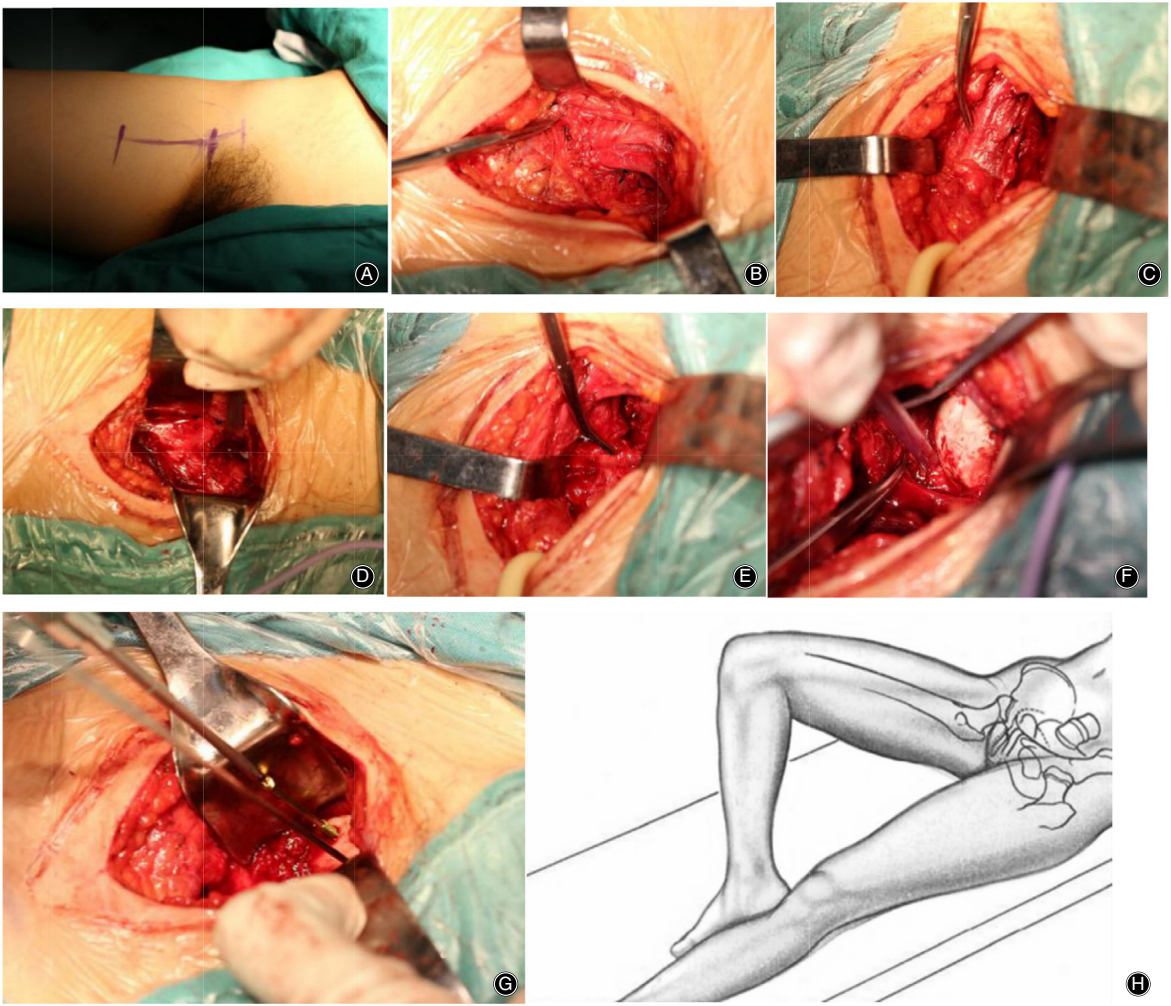
Anterior medial fenestration approach: (A) body surface location; (B) the medial margin of the femoral vessel exposes adductor longus and pectineus muscles; (C) femoral vascular sheath; (D) upward to deep blunt separation, the adductor group is pulled medially; (E) protect transverse branch of medial femoral circumflex artery below fenestration; (F) fully expose femoral head; (G) countersunk hollow nail fixed; (H) intraoperative position, with the patient taking the supine position. Flexion, abduction, and external rotation of the hip on the operative side.

The patient was placed in the supine position. Flexion, abduction, and external rotation of the hip was performed on the operative side. An incision of approximately 6–9 cm (1–2 cm above the head, 4–5 cm below the head, and 1–2 cm below the head) was made along the medial side of the femoral artery. The skin and subcutaneous tissue were incised and the deep fascia layer was opened. The deep fascia was incised and the femoral artery pulse was touched before exposing the adductor longus and pectineus at the medial edge of the femoral artery. Note the protection of the superficial great saphenous vein and its branches laterally. The gap between the adductor longus and pectineus muscle, which is at the medial edge of the pectineus muscle, was explored, and deep blunt separation was performed upward. After locating the lateral edge of the pectineus muscle, the pectineus, the short adductor, and the adductor longus were pulled together to the medial side. The blood vessels between muscles were ligated, as were the superficial external vessels of the pudendum when necessary. The femoral artery and the femoral vein were protected laterally, exposing the lesser trochanter. The iliac and psoas muscles were separated above the lesser trochanter and brought outward and upwards. The deep femoral artery and the transverse branch of the medial circumflex femoral artery were protected before bringing them down. The upper boundary exposed by the fenestration is the pubic body (the anterior lower part of the acetabulum). The lateral upper boundary is composed of the iliac muscle and the psoas major muscle. The lateral boundary is the rectus femoris and the femoral sheath, and the lower boundary is the deep femoral artery and the transverse branch of the medial circumflex femoral artery. The medial boundary is the pectineus muscle, the short adductor, and the adductor longus, and the pubic femoral ligament and iliofemoral ligament are revealed in fenestration. The capsule was opened and the pubic femoral ligament was pulled to the top, exposing the anterior inferior and anterior inferior quadrants of the femoral head (Fig. 1).

#### In Situ *Reduction and Fixation of the Femoral Head*

The blood in the joint cavity was cleared and the position was changed to reveal the quadrants of the femoral head, before exploring the displacement of the fractures of each quadrant. To fully expose the quadrants of the femoral head, the posture of the lower extremities can be changed; this can be achieved through the abduction, external rotation, adduction, internal rotation, and other activities of the hip joint, to check whether there are fractures in the rest of the femoral head.

Abductive external rotation position: Reveals most of the front outer lower part and part of the front inner lower quadrant.

Abductive external rotation, posterior extension: Reveals the front and outer upper, front outer and lower, front inner upper, and the front inner lower quadrant.

Abductive internal rotation position: Reveals most of the anterior outer upper quadrant and a small part of the anterior internal upper quadrant.

Abductive internal rotation, posterior extension: Reveals most of the front outer upper, front outer lower, and front inner upper quadrant.

The round ligament connected to the fracture block is not broken, the blood supply of the fracture block is preserved, and the fracture block is reduced *in situ*. The Kirschner wire was inserted for temporary fixation, and C‐arm fluoroscopy confirmed the reduction of the fracture blocks. After the satisfactory position was confirmed, a Countersunk hollow nail (Herbert hollow nail) was used for fixation. C‐arm fluoroscopy was performed again in order to confirm whether the screw length and fracture reduction were satisfactory. Intraoperative hip movement with abduction, adduction, internal rotation, external rotation, and extension was performed, and no hip dislocation or limitation of movement was observed. Finally, the wound was rinsed repeatedly to completely stop bleeding, and the joint capsule was sutured. A drainage tube was inserted into the incision and the incision was closed layer‐by‐layer.

#### *Perioperative Management*

Strict physical examination, X‐ray, and CT were performed in emergency cases, including clear sciatic nerve injury and posterior dislocation of the hip. Preoperative limb or bone traction, complete CT 3‐D reconstruction, MRI, and electromyography were used to clarify the acetabular, femoral head fracture quadrant, labrum glenoidale, and sciatic nerve injury. Timely treatment of combined injuries was ensured. No traction was performed after the operation, and anticoagulation was given on the second postoperative day. Hip rehabilitation training activities were performed on the bed, and coagulation indicators were closely monitored. Patients were allowed to sit up 1 week after surgery, perform non‐weight‐bearing training on the ground 1 month after surgery, and perform partial weight‐bearing training 2 months after surgery.

### *Observation Indexes*

All patients were treated using the anteromedial approach and were followed up regularly after surgery to observe and record the operation time, blood loss (Gross formula), follow‐up time, and last Thompson–Epstein evaluation^13^. The Harris score of the last follow up and the fracture healing time were recorded to evaluate the clinical efficacy.

#### *Blood Loss*

Gross formula: For assessing actual blood loss (ABL) in surgery. ABL = BV ×[Hcti‐Hctf]/Hctm. Total body volume (BV) = 70 mL × Body weight (kg). Hcti, before surgery; Hctf, end of surgery; Hctm, (Hcti + Hctf)/2.

#### *Thompson–Epstein Evaluation*

The postoperative efficacy was assessed, which included a hip function and X‐ray evaluation. The efficacy was deemed to be excellent if there was no pain and limited movement in the affected hip and if the X‐ray indicated that the hip joint had no fusion and no ossification, and the joint space was normal. The efficacy was deemed to be good if the hip joint motion was restored to 75% without pain and with limited movement, and the X‐ray indicated slight calcification of the joint capsule, narrowing of the joint space, and formation of osteophytes. The efficacy was determined to be fair in cases with limited hip movement, pain and discomfort, and when the X‐ray indicated moderate osteophyte formation, heterotopic ossification, femoral head collapse, and significantly narrowed joint space. Finally, poor efficacy was determined when there was obvious pain of the hip joint and limited movement, and the X‐ray indicated femoral head malunion, subchondral cystic change, a large amount of osteophyte formation, and severe narrowing of the joint space.

#### *Harris Hip Score*

The Harris hip score (HHS) was used to evaluate postoperative recovery of hip function in an adult population. The HHS score system mainly includes four aspects: pain, function, absence of deformity, and range of motion. The score standard had a maximum of 100 points (best possible outcome). A total score <70 is considered a poor score, 70–80 fair, 80–90 good, and 90–100 excellent.

### *Statistical Analysis*

All statistical analyses were carried out using SPSS statistical software version 22.0, and measurement data were tested for normal distribution using the Kolmogorov–Smirnov (KS) test. Measurement data were also tested using the homogeneity of variance test. Data with a normal distribution and equal variance are described as mean ± SD, and those without are described as median *M* and quartile *Q*_1_–*Q*_3_.

## Results

### *Anatomic Structures Related to the Anteromedial Fenestration Approach*

The femoral artery, the superficial external pudendal artery, and the deep femoral artery and its circumflex medial femoral artery can be seen in the anteromedial fenestration approach. The upper segment of the femoral vein exposed by this approach is located on the medial side of the femoral artery, and its upper branches include the great saphenous vein, the deep femoral vein, the superficial external pudendal and deep vein, the superficial epigastric vein, and the superficial circumflex iliac vein. The great saphenous vein, the external pudendal vein, and the deep femoral vein and its branches can be seen successively using the anteromedial fenestration approach. This approach does not expose the femoral nerve and its saphenous nerves. In the anteromedial fenestration approach, the femoral artery and vein and the great saphenous vein can be protected on the outside, the medial circumflex artery and vein can be protected on the bottom, and the superficial external artery and vein can be ligated if necessary.

**Figure 2:**
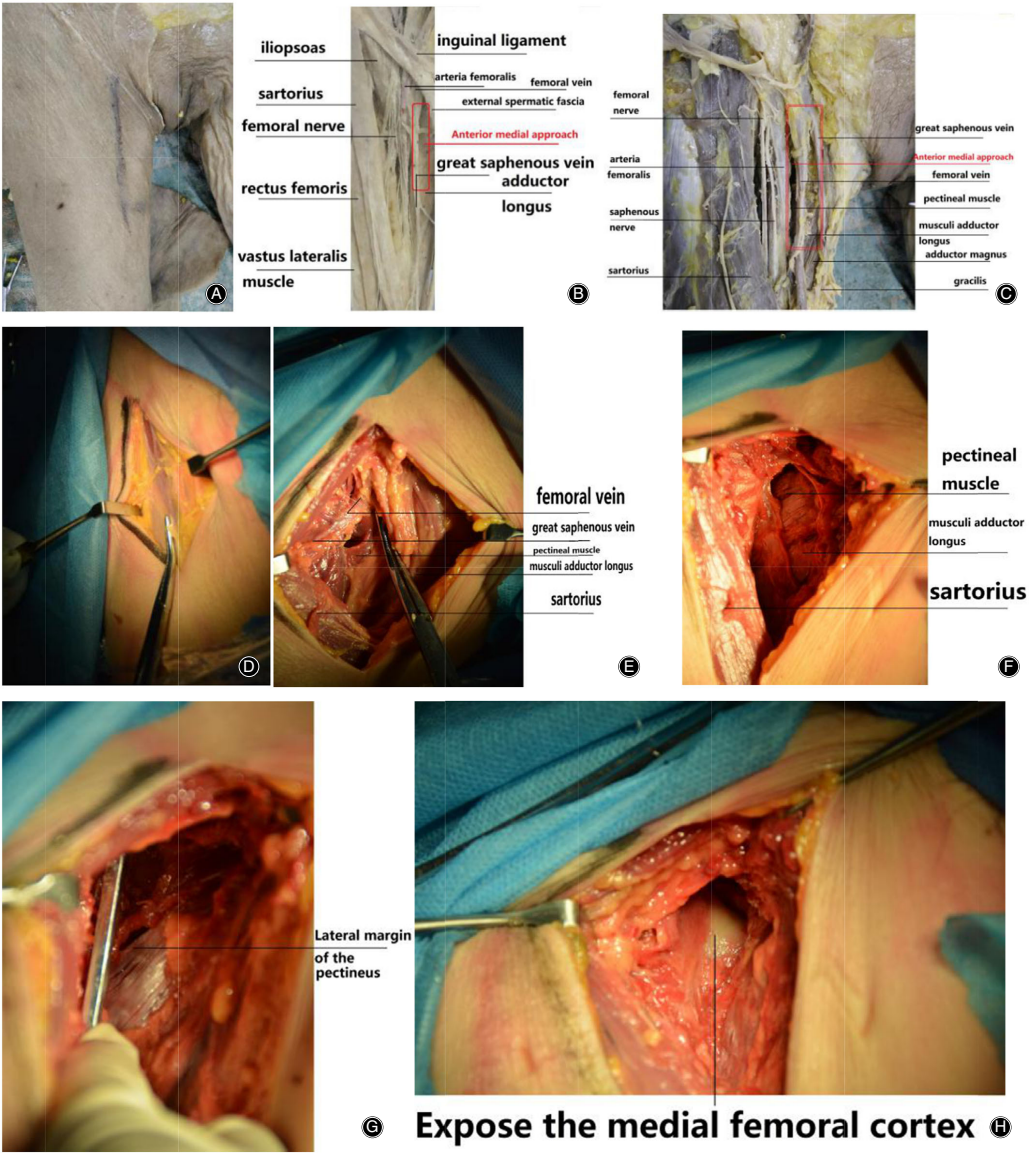
Anteromedial anatomy of the proximal femur: (A) anterior medial approach body surface marking; (B, C) description of the specific anatomical location of the anterior medial approach on the embalmed corpses treated by formalin; (D‐G) simulated anterior medial approach on fresh frozen corpses and related anatomical structures; and (H) the medial anterior approach exposes the medial wall of the femur and can expose the femoral head by upward blunt separation.

The adductor longus is the shallowest of the three adductors; it begins in front of the angle between the pubic crest and the pubic symphysis and ends in the middle of the thick line of the femur. The anteromedial fenestration approach only exposes the distal terminus of the psoas major and iliac muscles and passes down above the superior ramus of the pubis to the lesser trochanter of the femur. The anteromedial fenestration leads the adductor muscles (pectineus, short adductor, and adductor longus) to the medial lower side and the rectus femoris to the lateral side. The upper part of the fenestration is the pubic body (lower anterior acetabulum). The iliopsoas muscle is obtuse and separated and stretched outward to the upper part. The pubic ligament and the iliofemoral ligament are visible in the fenestration (Fig. 2).

#### *Surgical Approach Depth*

The straight‐line distance S_1_: 74–99 mm from the lower edge of the femoral head to the anterior medial approach (mean, 88.38 mm).

#### *Important Anatomical Depth*

The straight‐line distance from the transverse branch of the profunda artery and its branches and the medial circumflex artery to the incision skin S_2_: 59–89 mm (mean, 77.00 mm). The vertical distance from the plane of lesser trochanter to the plane of the femoral artery S_3_: 50–64 mm (mean, 57.25 mm).

#### *Plane Depth of Fenestration*

The linear distance S_4_: 72–95 mm from the lesser trochanter to the incision skin (mean, 84.25 mm, Table 1).

**Table 1:** Distance between four fresh frozen specimens and relevant anatomical structures of this approach

	Specimen 1: Female, 67 years		Specimen 2: Male, 75 years		Specimen 3: Female, 85 years		Specimen 4: Female, 78 years		Mean value	Median
Location	Right	Left	Right	Left	Right	Left	Right	Left		
S_1_ (mm)	84	83	99	97	76	74	98	96	88.38	90.00
S_2_ (mm)	76	77	89	88	64	59	84	79	77.00	78.00
S_3_ (mm)	50	53	60	58	54	57	62	64	57.25	57.50
S_4_ (mm)	82	83	86	89	75	72	92	95	84.25	84.50

### *Perioperative Period and Follow up*

In total, 12 cases of femoral head fracture were treated by anterior medial fenestration *in situ* reduction and fixation, including Pipkin type I (8 cases) and II (4 cases). The operation time was 96.25–118.75 min (median, 100 min), and the blood loss was 115.00 ± 22.76 mL. The follow‐up time was 18.58 ± 4.48 months, and the fracture healing time was 144.17 ± 14.53 days. The last Thomson–Epstein evaluation showed that 6 cases were excellent, 4 cases were good, and 2 cases were fair, with an excellent and good rate of 83.33%. The last Harris score was 85.08 ± 5.73 points. There were: no cases of wound nonunion and subcutaneous effusion; 1 case of ectopic ossification; 2 cases of traumatic osteoarthritis; and no cases of femoral head necrosis (Table 2).

**Table 2:** Data of 12 cases of femoral head fracture

Number	Pipkin type	Gender	Age (years)	Operating time (min)	Peri‐operative bleeding (mL)	Follow‐up time (months)	Fracture healing time (days)	The last Harris hip score (points)	The last Thompson–Epstein evaluation
1	I	F	38	95	100	12	120	93	Excellent
2	I	F	41	100	90	18	130	91	Excellent
3	II	F	45	110	140	15	150	81	Good
4	I	F	51	100	120	28	120	87	Excellent
5	I	M	53	90	85	21	130	88	Excellent
6	II	F	59	130	150	24	150	76	Fair
7	I	F	57	100	125	18	140	89	Excellent
8	II	F	48	120	135	15	160	82	Good
9	I	M	37	95	90	21	140	90	Excellent
10	I	F	52	115	100	20	150	85	Good
11	I	F	46	100	105	16	160	84	Good
12	II	M	55	135	140	15	180	75	Fair
^−^ *x* ± *s M* (Q_1_–Q_3)_	‐	‐	48.50 ± 7.27	100 (96.25–118.75)	115.00 ± 22.76	18.58 ± 4.48	144.17 ± 14.53	85.08 ± 5.73	Excellent and good rate 83.33%

F, female; M, male.

**Figure 3:**
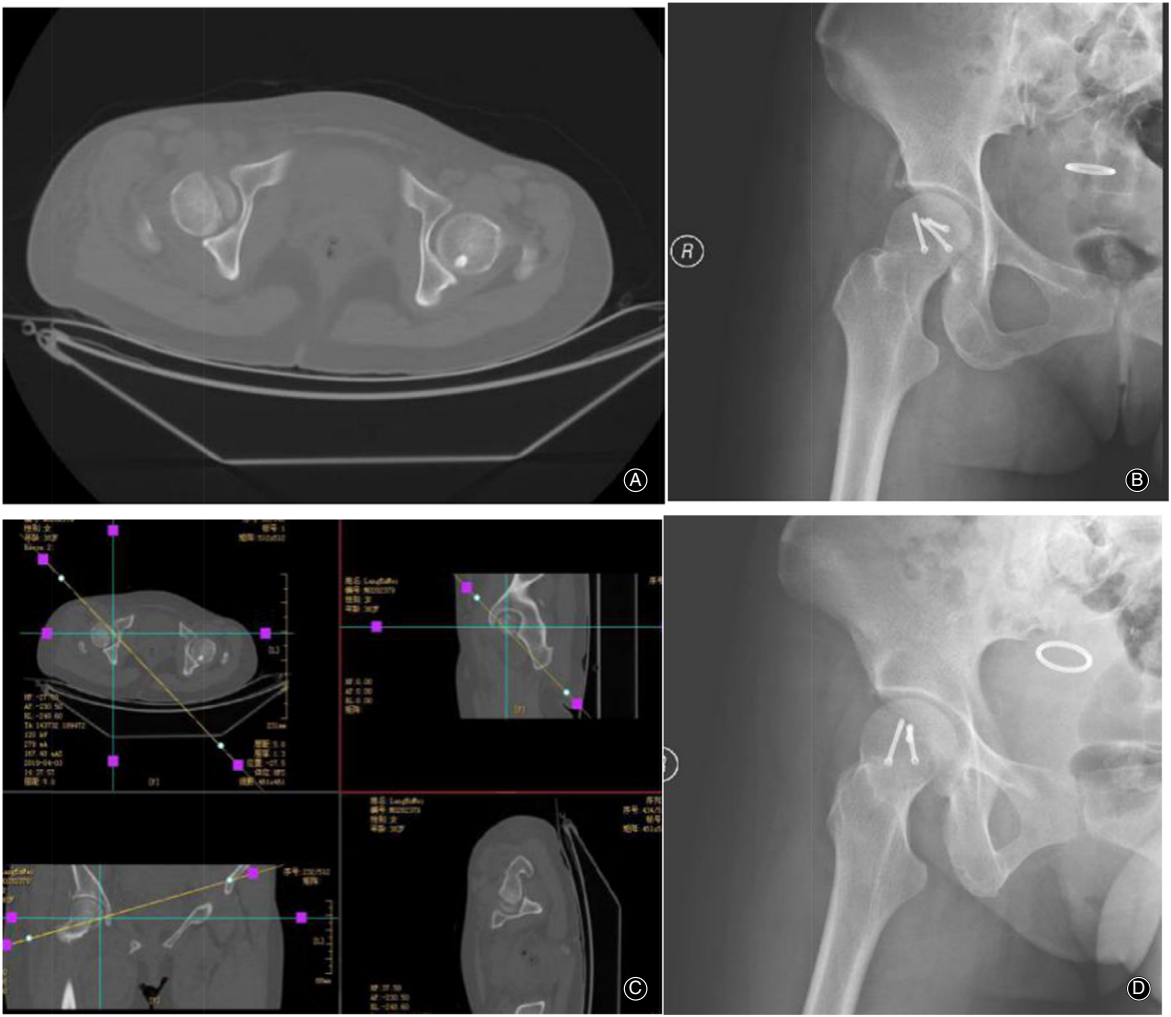
Femoral head fracture Pipkin II type imaging data: (A, B) preoperative CT and reconstruction; and (C, D) anterior and lateral X‐ray of the hip after operation.

For Pipkin type I cases, 8 had a good prognosis evaluation, with no hip pain or restricted movement, X‐ray demonstrating that the tip fractures had healed completely, and no narrow joint space (Fig. 3). A 55‐year‐old Pipkin type II female patient had a good prognosis; she complained of pain and discomfort in her hip when she squatted or stood for a long time. She was treated with non‐steroidal anti‐inflammatory (NSAID) drugs, and her symptoms were relieved. Her hip joint movement was slightly limited, and an X‐ray indicated that the joint space was slightly narrow. To avoid excessive weight‐bearing and movement of the affected limb, she was asked to strengthen the non‐weight‐bearing hip function with exercise, and at follow up, the fracture had completely healed.

A 59‐year‐old Pipkin type II female patient had a good prognosis; she complained of chronic pain and discomfort in her hip when she stood for a long time. She was treated with non‐steroidal anti‐inflammatory (NSAID) drugs and her symptoms were relieved. Soft tissue calcification around the hip joint, slightly limited abduction and rotation of the hip joint, increased pain when squatting, and no impact on daily activities were reported. An X‐ray indicated the formation of a small number of osteoblasts and narrowing of joint space. The patient was instructed to avoid excessive weight‐bearing and movement of the affected limb and asked to perform strengthening functional exercises for the non‐weight‐bearing hip and strengthening functional exercises for the quadriceps femoris, and to undergo nutritional joint treatment. The patient had no clinical symptoms of soft tissue calcification, no necrosis of the femoral head, and complete healing of the fracture upon follow‐up.

## Discussion

### *Particularity of Femoral Head Fracture and Significance of the Anteromedial Approach*

Studies have shown that femoral head fractures are complete intra‐articular fractures. The fracture block has no displacement, or the displacement is less than 2 mm, there is no fracture in the joint space, there are no cartilage fragments, the hip joint is stable, and the relationship between the femoral head and the hip glenoid is good. Conservative treatment can be followed in this situation. Cases of this type are more common in Pipkin type I and a small number of type II patients, and surgery is recommended for most other types of femoral head fractures^9^. The previous view for Pipkin I type fracture treatment was that conservative treatment after closed reduction should be chosen. With the deepening of research and understanding, a growing number of studies have shown that in fracture heads with a poor closed reduction and Pipkin type I fractures of the femoral head, surgery is still recommended. Epstein *et al*. suggested that all traumatic hip dislocation requires emergency surgical treatment, and that multiple attempts of closed reduction should be prohibited, as they believed that initial open reduction is better than initial closed reduction^13^. In addition, simple closed reduction and skin traction may lead to nonunion of the femoral head fracture, and with simple closed reduction it is difficult to achieve anatomical reduction of a femoral head fracture dislocation. Henle *et al*. reported that only 1 out of 12 patients had a correct fracture location after closed reduction^14^. Furthermore, Chakraborti *et al*. suggested conservative treatment after initial closed reduction^15^. According to Stewart *et al*., if the hip joint is not coordinated or stable after closed reduction, surgical treatment is required regardless of whether the head fragment is removed^16^. The Pipkin type I head below the debris is located in a central sunken non‐weight‐bearing area, and a fracture block of less than 1 cm can be removed^17^. Giannoudis *et al*. reviewed 29 cases of femoral head fracture and found that, in Pipkin type I damage, resection of the femoral head fracture block could restore hip joint function (86.7% for excellent or good)^18^. In recent years, researchers have demonstrated that the Pipkin type I also belongs to the intra‐articular fracture category and that femoral head fractures should be anatomically reduced. If central recess is required for larger pieces, open reduction and internal fixation is suggested^16^. The Pipkin type II block is located in the central concave type fractures mentioned above and involves a weight‐bearing area; in these cases, surgical reduction and fixation treatment are recommended.

There are many complications of femoral head fracture, including femoral head necrosis, ectopic ossification, traumatic osteoarthritis, and sciatic nerve injury^13^. Studies have shown that the incidence of traumatic osteoarthritis is 8%–75%, while the incidence of femoral head necrosis accounts for 6%–23%^19^. Anatomical reduction and fixation are important methods to reduce complications of femoral head fractures using a minimally invasive and small incision to reduce injury; they also avoid injury of the nutrient blood supply to the femoral head. The advantage of *in situ* reduction and fixation of the anteromedial fenestration approach lies in the minimally invasive fenestration approach in the muscular space that does not damage the blood supply of the femoral head, because the femoral vessels can be seen directly during the operation; this allows better protection of the femoral head below the lateral side of the fenestration. This approach is conducted in the medial part of the femoral sheath, far away from the femoral nerve, and the possibility of injury is greatly reduced compared to the anterior approach. Furthermore, the lateral femoral circumflex artery is not damaged because during the operation, the deep femoral artery and its branches, and the medial femoral circumflex artery, can be directly seen, which can be protected under fenestration, reducing the risk of femoral head necrosis. Another advantage of this approach is the *in situ* reduction. In previous approaches, the articular capsule is completely opened, and the round ligament of the femoral head is severed; at the same time, the round ligament artery of the femoral head is damaged, and the fracture is reduced and fixed outside the joint. This approach reduces fracture *in situ* without damaging the femoral round ligament artery. The feasibility of this approach was verified by anatomic study, and the depth of the surgical approach and important anatomical structures were measured. For Pipkin type I and II femoral head fractures, the femoral head fractures can be revealed by abduction, external rotation, internal rotation, and posterior extension of the hip joint to detect fracture displacement and to reset the fixation. It was clinically verified that postoperative hip function recovery was good, and the good and excellent rate was 83.33% in the last evaluation by Thomson–Epstein. However, because this was not compared with other approaches, it was impossible to verify whether the fracture healing time was shorter and there were fewer postoperative complications.

It is worth noting that the anterior medial approach is carried out very close to the femoral artery and vein. Once a deep infection occurs, it can be fatal and difficult to control. For this approach, the femoral vascular sheath is not opened and there is a low risk of infection. Fortunately, none of the 12 patients treated with the anteromedial approach developed infection. The disinfection of the operation area must be thorough, the aseptic operation standard should be strictly observed during the operation, and the operator should wear double aseptic gloves. Attention should be paid to the protection of femoral vessels during the operation, and antibiotics were routinely given intravenously within 24 h after the operation. After the operation, the inflammatory indexes were monitored, and the anti‐infection treatment was carried out. In addition, dressings were changed regularly to avoid wound infection.

### *Exposure and Key Points of the Anteromedial Approach of the Hip Joint*

The main point of this approach lies in revealing the fenestration and the exact search for fenestration. The difficulty lies in locating the adductor longus and the pectineus space, looking outward and deep for the lateral edge of the pectineus, while paying attention to protect the lateral femoral vessels when separating outward and performing blunt separation along the direction of the muscle fibers after opening the muscle fascia. It is important to protect the deep femoral artery and its branches and the medial circumflex femoral artery at the bottom of the fenestration. The obturator nerve must also be carefully protected. This approach is performed at the lateral margin of the pectineus; the anterior and posterior branches of the obturator nerve are, respectively, medial to the anterior and posterior parts of the short adductor muscle, with occasional branches to the pectineus muscle. The nerve is accompanied by the obturator artery, and it is important to remember that the surgical approach should be careful not to be too close to the side of the femoral vessels. The lesser trochanter is always taken as an important anatomical marker, and the direction of blunt separation is determined by the bone sign of the lesser trochanter, and the arteriovenous and direction of travel are distinguished by the blood vessel pulse from the fingers. After the capsule is cut open, the Hoffman retraction hook is inserted into the neck of the femur, and the iliopsoas muscle is pulled outward and above to expose the femoral head. After the fracture is reduced and fixed, the joint capsule is sutured, and the hip joint is activated to evaluate its function. It is also important to consider when to choose this particular approach, which is recommended for Pipkin type I and II femoral head fractures. This approach can also be selected for cases of anterior acetabular fractures or anterior labrum glenoidale injuries. The anterior medial approach combined with the posterior hip joint approach may also be used in patients with posterior acetabular fractures. Due to the limited exposure range of the anterolateral approach to the femoral head, it is difficult for the femoral head to break out of the joint capsule, and it is not recommended for cases with a large number of free small bone fragments in the joint space, as well as cases with comminuted fractures of the femoral head. This procedure is not recommended for Pipkin type I and II femoral head fractures with a posterior superior fracture and is also not recommended for patients with sciatic nerve injury. In the emergency department, strict physical examination was performed to determine the presence of hip dislocation and sciatic nerve injury.

CT, MRI, and EMG were timely completed before surgery^20^, ^21^, ^22^, and appropriate surgical approaches were selected according to the quadrant of the femoral head fracture, and acetabular, glenoid labrum, and sciatic nerve injuries.

### *Limitations*

The main limitation of the current study is the fact that there are few clinical cases of anteromedial fenestration approaches for femoral head fractures. Therefore, no comparative study has been conducted with the anterior approach, the anterolateral approach, or the greater trochanteric osteotomy approach; thus, the superiority of this approach cannot be fully validated as yet. The purpose of this study was to introduce an anterolateral approach to expose the quadrants of the femoral head through fenestration by changing the position of the limbs. The feasibility of this approach was demonstrated through anatomical study and clinical application. The next step is to increase the sample size, explore its advantages through comparative studies, extend the follow‐up time, and comprehensively evaluate the incidence of complications. Another deficiency of this study is that we only discuss the approach in the context of Pipkin type I and II femoral head fractures. Future research may be based on this approach combined with anterior wall acetabulum bone cutting and on increasing the exposed area of the femoral head to treat Pipkin type III and IV femoral fractures.
